# For the record: a narrative review of ambient voice technology in clinical documentation for dentistry and wider healthcare

**DOI:** 10.1038/s41415-026-9951-9

**Published:** 2026-09-25

**Authors:** Ruairí O´Kane, Daniel Stonehouse-Smith, Melody Shirazi, Rachel Hutchinson, Jadbinder Seehra, Martyn T. Cobourne

**Affiliations:** 761285122209519590762 Centre for Craniofacial Development & Regeneration, Faculty of Dentistry, Oral & Craniofacial Sciences, King´s College London, London, UK; Department of Orthodontics, Guy´s & St Thomas´ NHS Foundation Trust, United Kingdom; 464436150293787091369 Department of Orthodontics, Guy´s & St Thomas´ NHS Foundation Trust, United Kingdom

## Abstract

**Aims** We synthesise current evidence on ambient voice technologies (AVT) and generative artificial intelligence (AI) in dentistry and wider healthcare.

**Methods** Databases were searched across AI and AI-enabled clinical documentation, large language models and summarisation, and documentation outputs. Included studies evaluated AI-generation of clinical documentation with outcome measures including accuracy, documentation time, and the clinician/patient experience.

**Results** Evidence within dentistry is limited, with only one current study evaluating AVT speech-to-text accuracy. Performance varied between systems, with reduced accuracy for dental terminology and clinically significant errors and hallucinations observed. No dental studies evaluated AI-generation of clinical records or letters. Studies across wider healthcare reported variable performance, including errors with the potential to cause harm. Implementation studies suggest reductions in documentation time and burden with AVT; however, the magnitude of effect was inconsistent, with uptake varying between clinicians and note-length increased. Patient attitudes were favourable although conditional on the accuracy and privacy of using these tools.

**Conclusions** Ambient AI may reduce documentation time, but clinicians remain responsible for the final record produced by these systems. Ongoing research into safety and efficiency, alongside improved AI-literacy is required to ensure that the benefits of this technology are realised without compromising record accuracy or patient trust.

**Supplementary Information:**

Zusatzmaterial online: Zu diesem Beitrag sind unter https://doi.org/10.1038/s41415-026-9951-9 für autorisierte Leser zusätzliche Dateien abrufbar.

## Key points

- Digital technology in dentistry has improved conventional workflows; ambient voice technology (AVT) may automate documentation, but dental evidence remains limited.
- AVT has the capacity to reduce documentation time and time spent on clinical administration, but it can be associated with clinically significant errors. Clinicians remain responsible for the final clinical record.
- Patients may self-censor during recording of sensitive discussions. Notification that AVT is in use, with a meaningful opportunity to decline, is essential.

**Supplementary Information:**

Zusatzmaterial online: Zu diesem Beitrag sind unter https://doi.org/10.1038/s41415-026-9951-9 für autorisierte Leser zusätzliche Dateien abrufbar.

## Introduction

The move to electronic health records (EHR) has changed traditional methods of record-keeping in dentistry and wider healthcare; improving legibility and standardisation, but associated with more time-consuming and technical data-input and heavily keyboard-dependent, with copy-and-paste contributing to so-called ‘note bloat'.^1^ Across healthcare, documentation-burden has been linked to increased workload, clinician burnout and worse patient outcomes.^2^ Indeed, keyboard and computer-mediated interaction with EHR can intrude on the clinical encounter, reducing clinician eye-contact with the patient and impeding clinician-patient rapport.^3^ However, clear, concise and contemporaneous clinical records are mandatory for regulatory and professional bodies^4^^,^^5^ and there are clear advantages associated with reliable and efficient electronic documentation across healthcare.

**Fig. 1:**
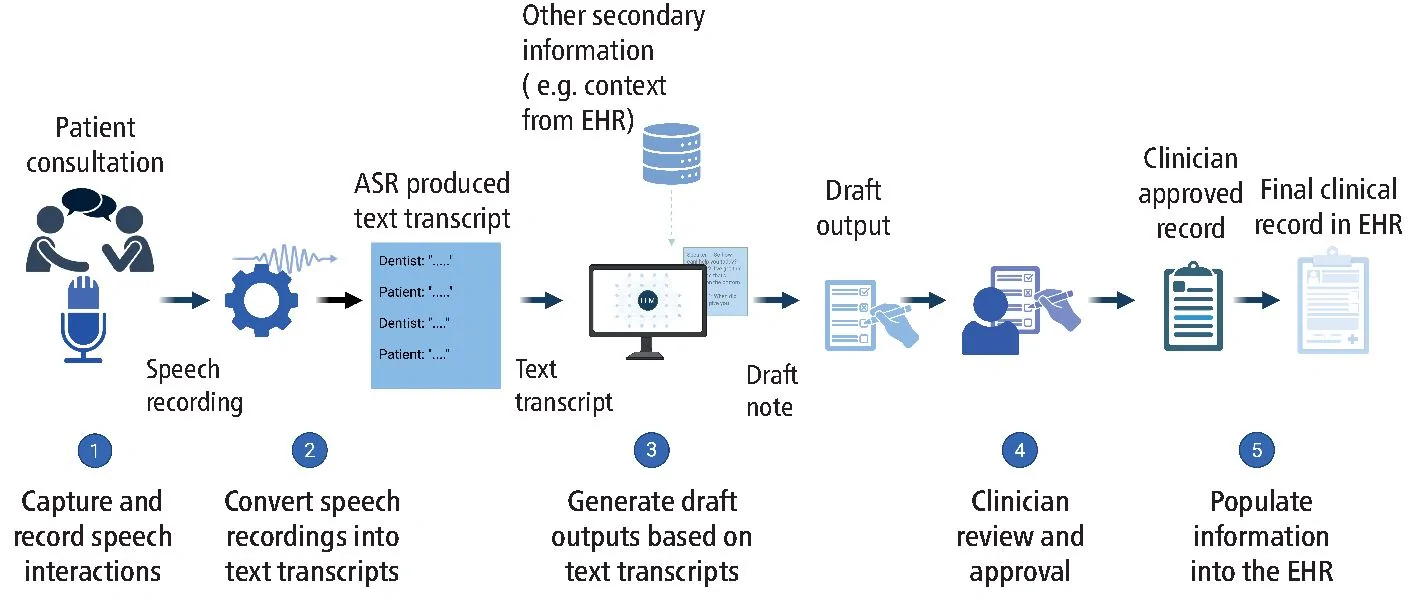
Overview of ambient voice technology during the clinician-patient consultation. (1) Speech interactions between the clinician and patient during a consultation are captured by a microphone; (2) Automatic speech recognition converts the recording into a diarised text transcript; (3) A large language model, potentially using existing electronic health record (EHR) context, generates a draft clinical note from the transcript; (4) The clinician reviews and approves the draft output; (5) The final record is populated into the EHR. Retention of audio recordings and transcripts varies by product, with automatic deletion preferred where appropriate. Created in https://BioRender.com and Adobe Photoshop 2026

Ambient artificial intelligence (AI) refers to AI embedded in the surroundings; sensing, interpreting and acting on contextual information with minimal user input.^6^ The most notable healthcare application has been in clinical documentation, where systems can autonomously capture the clinician-patient consultation and structure the clinical record. Ambient voice technologies (AVT), also referred to as digital scribes or AI scribes, use automatic speech recognition (ASR) to convert dictations or patient-clinician consultations into written text, with a large language model (LLM) then summarising and structuring the transcript into the clinical record (Fig. 1, Box 1). These systems are being advocated to replace keyboard and mouse in the clinic.^7^ Current National Health Service (NHS) England guidance on AI-enabled ambient scribing systems outlines risk assessment as a prerequisite for safe deployment of AVT.^8^ Beyond transcription and creation of the clinical notes, a single recording can generate multiple outputs including patient letters, referrals and notes using customisable templates, with future developments anticipated (Table 1).

**Table 1:** Current features and potential future developments of ambient voice technology in dentistry

**Area**	**Current features**	**Potential future developments**
Speech to draft note	Ambient recording of consultation or dictation with transcript generation Draft clinical note using templates/headings for clinician review	More reliable handling of dental terminology, background noise and overlapping speakers Uncertainty-aware speech recognition, with AI flagging clinically significant terms
Charting, structured data and imaging	Voice-assisted periodontal and restorative charting Limited integration to electronic health record (EHR); usually copy-and-paste	Bidirectional EHR integration (tooth chart, periodontal chart, treatment plan) Automatic incorporation of intraoral scans, CBCTs, imaging-AI findings into notes and charting Video-assisted charting from intra-oral images or video
Letters, referrals, consent and post-operative care	Patient letters and summaries in plain language Referral letters and replies Consent documentation with e-signatures Post-operative instructions and after-care advice	Multimodal patient summaries (text with annotated images or videos) Real-time clinician prompts when risks in consent have not been documented or discussed
Coding and claims	Procedure-code suggestions or text-to-code mapping	Automated mapping to clinical vocabularies (e.g., SNOMED CT) with clinician confirmation Auto-population and validation of administrative claim forms (e.g., NHS FP17)
Quality assurance and audit	Documentation audits against record-keeping standards (e.g., CGDent/FGDP) Standardised templates designed to reduce missing data items	Audit reporting for regulators or commissioners (e.g., CQC, NHS) Automated flagging of likely omissions or unsupported content in generated notes Practice-level documentation quality dashboards
Recall and continuity	Retrieval of prior notes or exams for recall appointments Editing and amending of previous notes for continuity	Automated ‘since last visit' summaries highlighting key changes and unresolved issues Longitudinal tracking of changes (e.g., periodontal indices, caries risk, bone levels)
Language and accessibility	Multilingual transcription or translation of patient-facing outputs	Real-time bidirectional translation during consultations (AI interpreter)
In-consultation prompts and decision support	Limited; some offer simple prompts based on entered findings (e.g., recall interval suggestions)	Prompts for undocumented essential information (e.g., allergies, consent, safeguarding flags) Medication interaction and antibiotic stewardship alerts Evidence-based clinical prompts
AI, artificial intelligence; CBCT, cone beam computed tomography; CGDent, College of General Dentistry; CQC, Care Quality Commission; EHR, electronic health record; FGDP, Faculty of General Dental Practice (UK); NHS, National Health Service; SNOMED CT, Systematized Nomenclature of Medicine Clinical Terms.		

Over the past decades, clinical dentistry has integrated multiple digital workflows into the clinical environment. Intra-oral scanning, cone beam computed tomography (CBCT) guided treatment planning, CAD/CAM (computer-aided design/manufacturing) milling and 3D printing have all required the profession to evaluate accuracy, establish standards and adapt the way these workflows perform. However, these technologies digitised existing processes; they changed how the task was performed and not whether the clinician performed it. AVT sits alongside radiographic analysis, patient triage and clinical decision support, and automated dental charting using video as part of a shift from digitisation to automation.^9^

Previous reviews in dentistry have focused on natural language processing (NLP) as a conduit for data extraction from dental records for research;^10^ however, evidence on AI to create the record itself, including earlier work on the digital scribe,^11^^,^^12^ has not been synthesised within the dental context. As dental professionals increasingly encounter these tools, this review aims to summarise current evidence on AVT and generative AI in dentistry and wider healthcare. Specifically, we focus on opportunities, challenges, practical implications and ethics, within the context of clinical documentation.

Box 1 Glossary of terms

- Ambient Voice Technology (AVT) – technology that passively captures patient-clinician conversations, generating draft clinical documentation and other content such as letters
- Automatic Speech Recognition (ASR) – the task of mapping a speech waveform to a corresponding sequence of words, representing the first step in AVT.
- Word Error Rate (WER) – metric quantifying the accuracy of ASR. It measures errors through substitutions (words that are mistaken), deletions (words not transcribed) and insertions (additional words) calculated relative to a reference transcript. A lower WER is better.
- Large Language Model (LLM) – a neural network trained on large quantities of text to predict the next word given a sequence of preceding words. This pretraining enables LLMs to generate, summarise and interpret natural language. Commercial products built on LLMs include ChatGPT (OpenAI), Gemini (Google) and Claude (Anthropic).
- Electronic Health Records (EHR) – digital systems used to record and share patient health information across clinicians and settings.
- Natural Language Processing (NLP) – A field of artificial intelligence and computational linguistics concerned with developing methods for analysing, representing, and generating human language.
- Definitions for ASR, WER, LLM and NLP are adapted from Jurafsky and Martin.^85^

## Methods

The PubMed, Embase, Medline and Cochrane Library databases were searched for publications from 1 January 2015 to 31 December 2025. This timeframe covers the emergence of cloud-based speech recognition and transformer architecture underpinning current documentation tools. Search terms combined MeSH headings, Emtree terms and key words across three areas:

- AI-enabled clinical documentation (e.g., speech recognition, ambient voice technology, scribe)
- LLM and summarisation
- Documentation outputs (e.g., notes, records, documentation, letters).

**Fig. 2:**
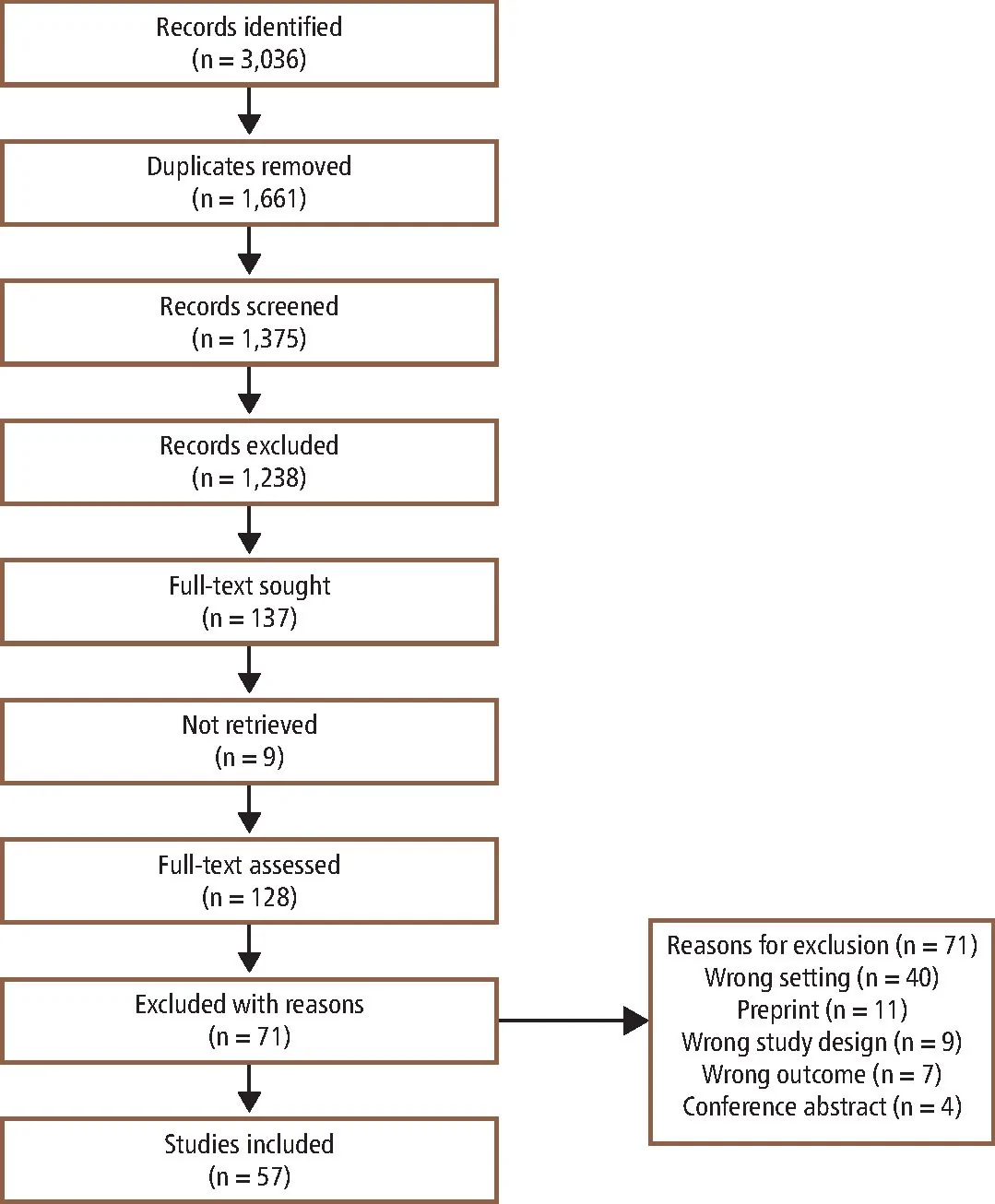
Flow diagram of the search and screening process.

Searches were run with and without dentistry-specific keywords to capture evidence from both settings with full search strings given in the online Supplementary Information. Inclusion criteria required studies to evaluate an AI-enabled approach to create, correct, structure or summarise clinical documentation and reported outcomes relating to accuracy, documentation quality, documentation time or clinician and/or patient experience. Studies using NLP to extract, classify or structure information from existing records or image analysis were excluded. In wider healthcare, inpatient or emergency department-only studies were excluded as outpatient and primary care settings are most transferable to dental practice. Two reviewers screened independently, with disagreements resolved by discussion. The search and screening process is summarised in Figure 2. Given the heterogeneity in settings, interventions, outcome measures and study designs, a narrative synthesis was used, and a risk-of-bias tool was not applied. Instead, study quality was considered during synthesis with attention to study design, setting, presence of a comparator or control, sample size, risk of confounding, outcome measurement approach, and declared conflicts of interest, with limitations noted where they affect conclusions.

## Results

**Fig. 3:**
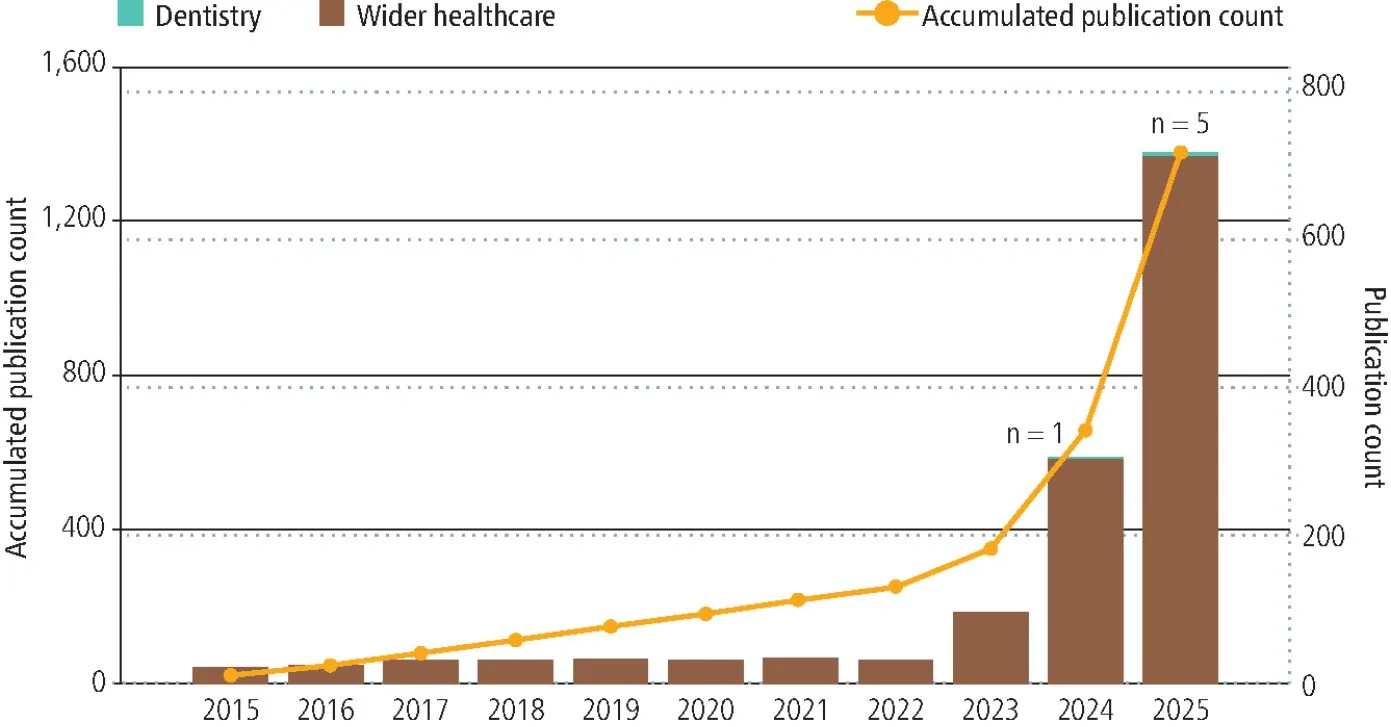
Annual and cumulative counts of ambient voice technology-related search results (2015–2025). Bars represent the number of publications per year in dentistry (light blue) and wider healthcare (brown) (right y-axis). The yellow line represents the cumulative publication count across both categories (left y-axis). Annotations indicate the number of search results within dentistry (n = 1 in 2024; n = 5 in 2025). Of the 57 studies included in the review, three evaluated outcomes within a dental setting

The search identified 3,036 studies, with 57 meeting the inclusion criteria. Most publications emerged from 2023 onwards, with studies in clinical dentistry only appearing from 2024 (Fig. 3). Included studies evaluated performance across the AVT workflow (speech to text; text to draft record; clinician review and approval) and the outcomes associated with implementation (documentation changes; consultation experience).

### Transcription accuracy: speech to text

ASR converts speech into text as the first step in ambient documentation. Accuracy is most commonly measured by using the word-error-rate metric (WER; lower is better), with the benchmark of professional human transcription achieving a WER of around 6%.^13^ General-purpose-ASR has improved,^14^ but challenges remain, particularly on clinical and technical vocabulary required for contemporary documentation.

Only one study has evaluated transcription accuracy within dentistry, comparing ten ASR systems using narrated orthodontic records as the experimental model. Performance varied substantially: a two-stage experimental ASR-LLM pipeline achieved a WER = 3.7%, surpassing the human benchmark and eliminating the gap between general and clinical terminology; whilst the best commercial system achieved a WER = 5.4%. Despite acceptable transcription of general language, all other products performed worse on dental terminology and clinically significant errors were seen across tools. Some systems inserted text not spoken (‘hallucinations') including one where an instruction to use a facemask for a minimum of 16 hours daily was transcribed as a minimum of ten minutes.^15^ Across healthcare settings, accuracy decreases in multi-speaker and noisy environments, with much variability between systems.^16^^,^^17^^,^^18^ Moreover, brief verbal responses such as ‘mm-hm' and ‘uh-uh', which can function as acknowledgment or implicit yes or no, were frequently misrecognised or omitted, producing clinically relevant distortions.^19^ Error rates were also higher among non-native English speakers and in multilingual settings.^20^ At the patient level, WERs were higher for Black patients than for white patients, with the magnitude varying by system.^21^

### Creating clinical documentation: text to record

Creating clinical documentation describes the step after transcription, where unstructured text is converted into the clinical record. Unlike transcription, which involves fidelity to what was spoken, selecting what is clinically relevant, organising it and determining what can safely be omitted in the clinical record requires judgment. AI-drafted notes read fluently, but omissions and hallucinations which are sufficient to change patient management can occur. There are no studies evaluating AI-generation of clinical notes or letters within dentistry. There is limited evidence evaluating AI-generated postoperative dental advice, with a content validity of 65.62%^22^ and AI-generated radiograph reports, which were shorter but contained fewer diagnostic findings than student-written reports.^23^

Outside dentistry, the evidence is mixed, spanning retrospective cohorts, simulation-studies and varied input types. When assessed using validated note-quality instruments such as the Physician Documentation Quality Instrument (PDQI-9), AI-generated notes score well for organisation and thoroughness and are often preferred by reviewers.^24^ In letter writing, AI-generated letters scored higher than manual-written letters,^25^^,^^26^ and AI-generated plain-language ophthalmology summaries improved patient understanding.^27^ Approval rates for notes were highest when clinicians co-drafted them with the use of AI.^28^^,^^29^ Across studies, evaluation methods varied, note-quality metrics and averaged Likert scores did not consistently align with clinical safety, with organisation and readability scoring higher than accuracy scores.^24^^,^^30^ Comparative evaluations of scribes showed variation between products, with recurring omissions, misplaced information and extraneous content. Amongst different scribes, only 35.8% of statements were consistent on the same encounter.^31^^,^^32^^,^^33^^,^^34^^,^^35^^,^^36^ with errors falling into two categories: hallucinations, where AI generates information not present in the source; and omissions, where clinically relevant content is lost. Omissions accounted for most errors, with reported rates suggesting hallucinations in 1.47% of note sentences and omissions in 3.45% of transcript sentences. Indeed, 44% of hallucinated sentences and 16.7% of omissions were classified as major harm; sufficient to change diagnosis or management of a patient if left uncorrected.^37^^,^^38^ When compared directly with clinicians, AI was less likely to omit information but more prone to extraneous detail and hallucinations.^39^

### Reviewing the draft record: clinician editing and approval

Across studies, AI-generated notes are positioned as drafts, and human review makes a meaningful difference; reducing clinically significant errors in ASR-generated notes from 63.6% to 7.8%.^40^ Simulation studies confirm that clinically consequential errors persist even in controlled conditions, and that omissions may be particularly difficult to identify because they require recognising what is absent rather than correcting what is present.^32^^,^^34^^,^^37^^,^^38^ However, measures of editing burden with these systems are uncommon. In simulation, clinicians required a mean 4.5 minutes to edit each draft note with time-to-final note varying by system.^33^^,^^34^ The user interface also contributes; in a comparison study, sign-in and launch required four to seven steps and most required copy-and-paste into the EHR. The initial draft was created in approximately a minute for a 15-minute encounter.^31^

### How documentation changes

Implementation studies commonly assess documentation-time using EHR activity metrics such as the time-in-notes and after-hours work. A pragmatic trial comparing two commercial scribes with usual care found modest time-in-note changes, with only one product outperforming usual care.^41^ Pre-post cohort evaluations using EHR telemetry generally reported reductions in note-writing time per note or appointment, though most lacked control groups.^42^^,^^43^^,^^44^^,^^45^^,^^46^^,^^47^^,^^48^ Uptake varied substantially between clinicians and time-savings were clinician-dependent.^44^^,^^49^ In one study, evidence suggested that benefits were not immediate, with after-hours documentation increased initially with use but then decreased by the end of the study, albeit alongside human scribe review.^50^

Interestingly, several implementation studies reporting reductions in time-in-notes were accompanied by increases in note length,^42^^,^^51^ with EHR telemetry showing this increase while the proportion of text typed by clinicians decreased.^20^^,^^43^^,^^45^^,^^47^ Financial productivity-linked outcomes were reported less frequently. In a hybrid model combining ambient scribe with human scribe review, adoption showed a 12.1% increase in the productivity metric, work relative value units.^50^ Barriers to continued use included lack of EHR integration, difficulty with multiple speakers and concerns about increases in note length.^52^

Self-reported burden and burnout decreased in most implementation studies.^46^^,^^51^^,^^53^^,^^54^^,^^55^^,^^56^^,^^57^ In the largest multi-site survey, the proportion of clinicians meeting the criteria for burnout on the Professional Fulfillment Index fell from 50.6% to 29.4%.^53^ Findings were not uniform, with one study finding no statistically significant changes and another reporting improved task load and documentation experience without significant changes in burnout.^58^ Even where documentation improved, users reported concerns about variable note quality, increasing note length and ongoing proofreading.^47^^,^^55^^,^^59^

### How the consultation changes

Patients were generally favourable towards AVT, but favourability fell when fuller explanations were given, becoming conditional on accuracy and privacy.^60^^,^^61^ In a pre-implementation survey (n = 1,893 patients), 48% were favourable, 33% neutral and 19% unfavourable.^60^ Consent rates proved sensitive to framing: brief explanations were associated with higher consent rates (81.6%), whereas expanded explanations detailing data storage and corporate involvement, reduced this to 55.3%. Some patients even reported they would censor their speech during discussions when AVT were in use.^62^ Clinicians reported benefits to patient engagement and undivided attention.^47^^,^^63^^,^^64^ However, concerns about consent, data security and output inaccuracies were common.^35^^,^^64^^,^^65^^,^^66^^,^^67^^,^^68^^,^^69^^,^^70^ Among general practitioners, only 66% had read terms and conditions and only 59% routinely sought patient consent.^65^

## Discussion

In dentistry, the adoption of AVT may be outpacing evaluation with only three studies within dentistry being found and none of them evaluating the full AVT workflow. The evidence within wider healthcare is larger but heterogenous. Documentation time and self-reported burden decreased most consistently across studies, with moderate confidence in the evidence of this direction of effect. However, the magnitude of this effect is harder to establish. Most evidence comes from uncontrolled pre-post designs with outcome measures that frequently rely on proxies such as user activity metrics and self-reported experience. Reduction in time is likewise accompanied by longer notes, variable clinician uptake, and some patients reporting that they would self-censor sensitive conversations. Assessments of accuracy and clinical risk are less common, and generated records can omit and hallucinate clinical details sufficient to change patient management with performance on clinical terminology shown to be lower. Yet this evidence derives predominantly from simulation studies and single-site evaluations with controlled comparative evaluations remaining rare and as a result, confidence in the evidence low. Consequently, it remains difficult to separate intrinsic system performance from how AVT is used, how users are trained and how it integrates into the environment of the EHR. Translating evidence from wider healthcare is challenging given dentistry's distinct vocabulary and background noise.

Before evaluating what AVT might improve, it is worth considering what it will replace. Records in dentistry are frequently incomplete or inaccurate, with variation in the correctness and completeness of charting.^71^^,^^72^ The gap between what is discussed and what is documented may be larger than generally thought. In nursing, 50.5% of patient problems and 20.8% of interventions discussed verbally were not documented.^73^ This reframes AVT as, in part, a response to time-pressure leading to under-documentation. However, capturing more of the encounter does not necessarily produce a better record.^74^ The accuracy of AVT depends on two sequential steps, each introducing risks: transcription of speech into text, and summarisation of text into the record. At the transcription step, low average error rates can conceal clinically significant errors involving negation, tooth identifiers and diagnoses, with nearly all systems performing worse on dental terminology.^15^ Accuracy degrades further in the noisy, multi-speaker, conversational setting of a dental practice,^15^^,^^16^^,^^17^ and appears lower for people with speech impairment, dementia, non-native English speakers and in multilingual settings.^20^^,^^75^ Differences across patient demographics raise equity concerns, underscoring the need for diverse, dental-specific training data.^21^

At the summarisation step, the evidence within dentistry is largely absent. In wider healthcare, Physician Documentation Quality Instrument (nine-item version) and similar validated tools, suggest generated notes can read well and may be preferred by reviewers, yet these surface measures are insufficient for assessing safety.^24^ Evaluation should therefore move beyond Likert-based ratings towards assessments grounded in the clinical significance of inaccuracies, omissions and hallucinations. Whether increases in note length associated with AVT, improve the capture of clinically relevant information or exacerbate ‘note bloat', remains unresolved.^20^^,^^42^^,^^47^^,^^51^ If the latter, this may drive the adoption of EHR-integrated LLM chart summaries, adding another layer at which clinical details may be omitted or distorted, with evaluation of such tools already emerging.^76^

By changing the mechanics of documentation, AVT shifts the role of the clinician from ‘author to editor'.^77^ Editing a draft may reduce cognitive load^58^ and afford more attention to the patient but it also displaces one form of labour with another. This shift is not only practical but epistemological. A clinician-authored note, however imperfect, reflects their clinical reasoning and interpretation of the encounter whereas an AI-generated note introduces a version of the consultation authored by neither party present, constructed from statistical patterns rather than clinical judgment. A challenge is that the most consequential errors such as omissions can be difficult to catch because they require recognising what is absent. Dentistry introduces the possibility of numerical substitutions, laterality or tooth-notation errors (e.g., ‘LR6' versus ‘LL6'), where limited semantic context and reliance on acoustic cues, combined with phonetic similarity between /l/ and /r/, makes clinically significant mistranscription possible, particularly in noise.^15^^,^^78^ As apparent fluency improves, the risk of automation bias grows; a fluent note containing a significant error may receive less scrutiny than a rough draft. As clinicians retain responsibility for the final record, they must guard against automation bias to avoid becoming ‘liability sinks' for AI.^79^

Reported reductions in documentation time and burnout, drawn from a growing number of commercial product evaluations in wider healthcare,^80^ represent perhaps the strongest case for AVT adoption, with the direction of effect favourable across most studies. However, these findings should be interpreted with some caution. The reliance on user activity metrics such as ‘time-in-note' may overestimate benefit. Time savings were more consistently favourable in studies that declared financial ties to the product under evaluation, whereas studies without such ties demonstrated more heterogenous findings. Variation in clinician uptake suggests that AVT may substantially benefit some users and not others,^42^^,^^45^^,^^47^ and comparative evaluations found substantial variation between products in omissions, hallucinations and editing burden.^31^^,^^32^^,^^33^^,^^34^ In dentistry, where no study has evaluated the full AVT workflow, time savings may be greater for consultation and treatment planning than for treatment appointments. Whether this time saving will reduce after-work documentation time or convert into greater capacity with shorter appointments and more patients remains unknown.

Patient attitudes towards AVT are generally favourable, but with two caveats. First, consent rates fell when patients received fuller explanations of how their data would be used, suggesting favourability may partly reflect incomplete understanding rather than informed acceptance.^60^^,^^62^ Secondly, the willingness of some patients to censor their speech during sensitive discussions raises concerns that reduced candour may degrade communication and patient care. Clinicians report similar governance concerns around consent, data security and inaccuracies.^65^^,^^66^^,^^67^ Adoption should improve patient-clinician interaction without reducing transparency or making opting-out difficult for patients.

Documenting care is a key part of providing it, with implied consent extending to the record. The move from pen to keyboard changed how the record was made, but not who made it; AVT has changed this with a third party now capturing and processing the consultation. NHS England guidance does not require explicit consent for AVT in direct care, provided that patients are notified and have a right to object under General Data Protection Regulation.^8^ The weight therefore falls on how patients are informed and if notification is cursory or inaccessible, this right to object becomes nominal.

Safe implementation of AVT depends on effective governance at organisational, regulatory and professional levels. NHS England guidance addresses clinical risk management, data protection and ongoing monitoring, with clinical safety standards applying to both organisations (DCB0160) and suppliers (DCB0129). The NHS has recently published a self-certified supplier registry covering AI scribes (https://digital.nhs.uk/services/ambient-scribing/ambient-voice-technology-self-certified-supplier-registry). Data processing and retention policies differ by product, with automatic deletion recommended where appropriate.^8^ Prompt-injection attacks, such as those subverting vision-language models in oncology, highlight the importance of cyber-security.^81^ How clinicians interact with the EHR shapes clinical behaviour; a cohort study of 2.5 million prescriptions found that design differences between EHR systems strongly influenced prescribing safety.^82^ As AVT integration with the EHR deepens (Table 1), implementation requires AI literacy. GDC-commissioned work has highlighted the absence of best-practice guidance for Dental AI deployment and the need for explicit training expectations.^83^ Table 2 maps the key risks and mitigation requirements at each stage of the AVT-assisted documentation process.

**Table 2:** Risks, mitigation and monitoring requirements for clinicians across stages of the ambient voice technology use in dentistry

**Stage of ambient voice technology-assisted documentation process**	**Risks**	**Mitigations**	**What to monitor**
Capturing and recording speech interactions	Privacy: background conversation in open bays; radios; inadvertent recording of others Behavioural: Reduced patient candour regarding sensitive discussions e.g., safeguarding Technical: poor microphone placement or quality Security: device loss/theft Data location: uncertainty about where audio is processed and stored Process: uncertainty regarding when a recording should pause or restart	Hardware: Lavalier microphones or directional microphones with clear placement guidance, test in the surgery before use Clear process for consent: verbal script, record only after patient has been informed and given the opportunity to decline, staff training Clinic layout: Designated bays where recording should occur, prefer background music on radios to spoken radio. Data processing: review terms and conditions, confirming where data are processed and whether recordings are retained Sensitive discussions: define when AVT is not used (e.g., high anxiety conversations, safeguarding disclosures)	Notification and decline rates, how often patients decline and reasons Patient feedback on privacy/candour/experience when using AVT Reported incidents including issues with consent or where audio from other conversations is captured
Converting speech recordings into text transcripts	Clinically significant mistranscriptions: tooth notation and laterality, negation Numbers: doses, units, wire dimensions, 6-point pocket chart (6PPC) Hallucinations: words not spoken being inserted Conversational cues lost: ‘mm-hm/uh-uh', interrupted speech, omissions Attribution errors: speaker diarisation errors (patient vs clinician) Noise: variable decrease in performance related to background noise Equity and bias: higher error rates for non-native speakers and for specific patient groups	Document using two different methods for tooth identifiers: i.e., using alphanumeric LR6 and ‘lower right first permanent molar' Review: numbers, tooth identifiers, laterality, doses, units, 6PPC Paraphrasing: restate key details to confirm accuracy Background noise: Minimising background noise, avoid concurrent spoken audio (radio/other conversations), if background noise remains too high, use typed documentation	Recording errors: record keeping audit to measure the clinically significant mistranscriptions in unedited notes and edited notes Fairness: are error rates consistently higher for specific patient groups?
Generating draft outputs based on text transcripts	Hallucinations: adds findings, diagnoses, consent, discussion Omissions: misses key negatives, consent/advice/safety-netting, relevant medical history Misstructuring of the note: correct detail placed in the incorrect section of the note Note bloat: extraneous, unnecessary detail included	Draft note: treat all outputs as a draft Templates: use of templates within the AVT that match your own documentation	Hallucination rates Omissions: what details are left out of notes Length of notes
Clinician review and approval	Automation bias: missed errors as the note looks plausible Time taken to review notes may reduce time savings from use of AVT	Checklist: verify tooth identifiers, laterality, diagnosis, consent, medication, units, and any safety-netting including omissions	Review editing time: record how long it takes to review and edit the note Log errors that were not found by clinician review and persisted in the note
Populate information into the Electronic Health Record	Copy and paste errors: information pasted into the incorrect patient record Structured data errors: incorrect coding, wrong tooth mapped into the chart	Patient-identifier check: ensure patient identifiers are being checked before pasting into the correct note Review auto-populated fields	Log wrong-chart incidents and near-misses Record how often incorrect codes are inserted
AVT, ambient voice technology; 6PPC, six-point pocket chart.			

Beyond governance, adoption requires trust: clinicians trusting the tools and patients trusting that technology serves their interests. Compared with wider healthcare, the evidence base within dentistry is lacking. This is both a limitation and opportunity, requiring well-designed research addressing safety and implementation. The transformer architecture underpinning modern generative AI was introduced in ‘Attention is all you need' in 2017 and is among the most cited papers of the 21^st^ century.^84^ AVT may help return attention to patients and automate notetaking, but for now, our attention is still needed to ensure the record remains accurate.

## Conclusions

AVT represents a shift in dentistry from digitising existing processes to automating them and there is promising evidence from wider healthcare that AVT can reduce documentation-time. However, these gains depend on how AVT is integrated within the EHR, patient and clinician uptake, and the time required to review and edit draft notes produced by these systems. Clinicians remain responsible for the final record produced by AVT and implementation requires vigilance against automation bias and monitoring of errors alongside clear processes for informing patients, data governance and cyber-security. As AVT use expands, ongoing research of safety and efficiency, alongside improved AI-literacy, is required to ensure its benefits are realised without compromising record accuracy or patient trust.

## Supplementary Information

Supplementary information (DOCX 19KB)

## Data Availability

All data supporting the findings of this study are available within the paper, its references and the supplementary information.
