# Supplementary material for: For the record: a narrative review of ambient voice technology in clinical documentation for dentistry and wider healthcare
Source: Br Dent J. 2026 Sep 25;241(6):385–93. doi: 10.1038/s41415-026-9951-9 (PMC13614888; doi:10.1038/s41415-026-9951-9)
Supplement: Supplementary file 1 [file 41415_2026_9951_MOESM1_ESM.docx]

**Search Strings**

Search strategies used for each database are shown below. The date of the last search was 7^th^ January 2026. For each database, a wider healthcare search was conducted first, followed by a dentistry search that applied the same strategy with an additional dental subject filter.

**MEDLINE (Ovid)**

**Wider healthcare**

1. Speech Recognition Software/

2. (automatic speech recognition or ASR or speech recognit* or speech-to-text or "speech to text" or voice-to-text or "voice to text" or voice recognit*).ti,ab,kf.

3. (dictat* adj2 (speech recognit* or ASR or speech-to-text or voice recognit*)).ti,ab,kf.

4. ((ambient adj2 (voice or documentation or scribe* or scribing or technolog*)) or "ambient voice technolog*" or "ambient documentation" or "ambient scribing" or "ambient clinical intelligence").ti,ab,kf.

5. ("digital scribe*" or "ambient scribe*" or "AI scribe*" or "clinical documentation assistant*" or "documentation assistant*" or "automated documentation" or "documentation automation").ti,ab,kf.

6. 1 or 2 or 3 or 4 or 5

7. Large Language Models/ or Generative Artificial Intelligence/

8. (large language model* or LLM* or "generative AI" or chatgpt or "chat gpt" or gpt-4 or gpt-3 or gpt-4o or "generative pretrained transformer*" or transformer-based).ti,ab,kf.

9. (summari* or summariz* or summaris* or "text summarization" or "text summarisation" or "note generation" or "clinical note generation" or structur* or "structured note*" or "draft note*" or "automated note*" or "auto-generated note*").ti,ab,kf.

10. (7 or 8) and 9

11. 6 or 10

12. Electronic Health Records/ or Medical Records Systems, Computerized/ or Medical Records/

13. (electronic health record* or electronic medical record* or EHR or EMR).ti,ab,kf.

14. ("clinical documentation" or (clinical adj2 note*) or (progress adj2 note*) or (encounter adj2 note*) or (visit adj2 note*) or (discharge adj2 summar*) or (clinic adj2 letter*) or (referral adj2 letter*) or (patient adj2 record*) or (medical adj2 record*)).ti,ab,kf.

15. 12 or 13 or 14

16. 11 and 15

17. limit 16 to (english language and yr="2015 - 2025")

**Dentistry search**

Lines 1–16 as above, with additional dental filter:

18. exp Dentistry/ or exp Dental Records/ or exp Orthodontics/ or exp Periodontics/ or exp Endodontics/ or exp Surgery, Oral/

19. (dentistry or dental or dentist* or orthodontic* or periodontic* or endodontic* or "oral health" or "oral surgery" or maxillofacial or "dental hygien*").ti,ab,kf.

20. 18 or 19

21. 16 and 20

22. limit 21 to (english language and yr="2015 - 2025")

**Embase (Ovid)**

**Wider healthcare**

1. exp speech recognition/ or exp speech recognition software/

2. (automatic speech recognition or ASR or speech recognit* or speech-to-text or "speech to text" or voice-to-text or "voice to text" or voice recognit*).ti,ab,kw.

3. (dictat* adj2 (speech recognit* or ASR or speech-to-text or voice recognit*)).ti,ab,kw.

4. ((ambient adj2 (voice or documentation or scribe* or scribing or technolog*)) or "ambient voice technolog*" or "ambient documentation" or "ambient scribing" or "ambient clinical intelligence").ti,ab,kw.

5. ("digital scribe*" or "ambient scribe*" or "AI scribe*" or "clinical documentation assistant*" or "documentation assistant*" or "automated documentation" or "documentation automation").ti,ab,kw.

6. 1 or 2 or 3 or 4 or 5

7. (large language model* or LLM* or "generative AI" or chatgpt or "chat gpt" or gpt-4 or gpt-3 or gpt-4o or "generative pretrained transformer*" or transformer-based).ti,ab,kw.

8. (summari* or summariz* or summaris* or "text summarization" or "text summarisation" or "note generation" or "clinical note generation" or structur* or "structured note*" or "draft note*" or "automated note*" or "auto-generated note*").ti,ab,kw.

9. 7 and 8

10. exp electronic health record/ or exp electronic medical record/ or exp medical record/

11. (electronic health record* or electronic medical record* or EHR or EMR).ti,ab,kw.

12. ("clinical documentation" or (clinical adj2 note*) or (progress adj2 note*) or (encounter adj2 note*) or (visit adj2 note*) or (discharge adj2 summar*) or (clinic adj2 letter*) or (referral adj2 letter*) or (patient adj2 record*) or (medical adj2 record*)).ti,ab,kw.

13. 10 or 11 or 12

14. (6 or 9) and 13

15. limit 14 to (english language and yr="2015 - 2025")

**Dentistry search**

Lines 1–15 as above, with additional dental filter:

16. exp dentistry/ or exp dental record/ or exp orthodontics/ or exp periodontics/ or exp endodontics/ or exp oral surgery/

17. (dentistry or dental or dentist* or orthodontic* or periodontic* or endodontic* or "oral health" or "oral surgery" or maxillofacial or "dental hygien*").ti,ab,kw.

18. 16 or 17

19. 15 and 18

20. limit 19 to (english language and yr="2015 - 2025")

**PubMed**

**Wider healthcare**

Concept 1: AI-enabled clinical documentation

("Speech Recognition Software"[Mesh]

OR "automatic speech recognition"[tiab] OR ASR[tiab] OR speech recognit*[tiab]

OR "speech-to-text"[tiab] OR "speech to text"[tiab]

OR "voice-to-text"[tiab] OR "voice to text"[tiab] OR voice recognit*[tiab]

OR ambient voice technolog*[tiab] OR "ambient documentation"[tiab] OR "ambient scribing"[tiab]

OR "ambient clinical intelligence"[tiab]

OR digital scribe*[tiab] OR ambient scribe*[tiab] OR AI scribe*[tiab]

OR clinical documentation assistant*[tiab] OR documentation assistant*[tiab]

OR "automated documentation"[tiab] OR "documentation automation"[tiab]

OR (

("Large Language Models"[Mesh] OR "Generative Artificial Intelligence"[Mesh]

OR "large language model*"[tiab] OR LLM[tiab] OR LLMs[tiab] OR "generative AI"[tiab]

OR ChatGPT[tiab] OR "Chat GPT"[tiab] OR GPT-4[tiab] OR GPT-3[tiab] OR GPT-4o[tiab]

OR generative pretrained transformer*[tiab] OR transformer-based[tiab])

AND

(summari*[tiab] OR summariz*[tiab] OR summaris*[tiab]

OR "text summarization"[tiab] OR "text summarisation"[tiab]

OR "note generation"[tiab] OR "clinical note generation"[tiab]

OR structur*[tiab] OR structured note*[tiab]

OR draft note*[tiab] OR automated note*[tiab] OR auto-generated note*[tiab])

))

AND

Concept 2: Documentation outputs

("Electronic Health Records"[Mesh] OR "Medical Records Systems, Computerized"[Mesh] OR "Medical Records"[Mesh]

OR "clinical documentation"[tiab]

OR clinical note*[tiab] OR progress note*[tiab] OR encounter note*[tiab] OR visit note*[tiab]

OR discharge summar*[tiab] OR clinic letter*[tiab] OR referral letter*[tiab]

OR electronic health record*[tiab] OR electronic medical record*[tiab] OR EHR[tiab] OR EMR[tiab]

OR patient record*[tiab] OR medical record*[tiab])

AND ("2015/01/01"[dp] : "2025/12/31"[dp])

AND English[lang]

NOT (animals[mh] NOT humans[mh])

**Dentistry search**

The above search with additional dental filter:

AND

("Dentistry"[Mesh] OR "Dental Records"[Mesh] OR "Orthodontics"[Mesh]

OR "Periodontics"[Mesh] OR "Endodontics"[Mesh] OR "Surgery, Oral"[Mesh]

OR dentistry[tiab] OR dental[tiab] OR dentist*[tiab] OR orthodontic*[tiab]

OR periodontic*[tiab] OR endodontic*[tiab] OR "oral health"[tiab]

OR "oral surgery"[tiab] OR maxillofacial[tiab] OR dental hygien*[tiab])

**Cochrane Library**

**Wider healthcare**

1. [mh "Speech Recognition Software"]
2. (automatic NEXT speech NEXT recognition OR ASR OR "speech recognition" OR "speech-to-text" OR "speech to text" OR "voice-to-text" OR "voice to text" OR "voice recognition"):ti,ab,kw
3. (ambient NEXT voice NEXT technolog* OR ambient NEXT documentation OR ambient NEXT scribing OR ambient NEXT clinical NEXT intelligence OR digital NEXT scribe* OR ambient NEXT scribe* OR AI NEXT scribe* OR clinical NEXT documentation NEXT assistant* OR documentation NEXT assistant* OR automated NEXT documentation OR documentation NEXT automation):ti,ab,kw
4. ("large language model" OR "large language models" OR LLM* OR "generative AI" OR ChatGPT OR "Chat GPT" OR GPT-4 OR GPT-3 OR GPT-4o OR "generative pretrained transformer" OR "generative pretrained transformers" OR "transformer-based"):ti,ab,kw
5. (summari* OR text NEXT summar* OR note NEXT generat* OR clinical NEXT note NEXT generat* OR structur* OR structured NEXT note* OR draft NEXT note* OR automated NEXT note* OR auto-generated NEXT note*):ti,ab,kw
6. #4 AND #5
7. [mh "Electronic Health Records"] OR [mh "Medical Records Systems, Computerized"] OR [mh "Medical Records"]
8. ("electronic health record" OR "electronic health records" OR "electronic medical record" OR "electronic medical records" OR EHR OR EMR OR "clinical documentation" OR clinical NEXT note* OR progress NEXT note* OR encounter NEXT note* OR visit NEXT note* OR discharge NEXT summar* OR clinic NEXT letter* OR referral NEXT letter* OR patient NEXT record* OR medical NEXT record*):ti,ab,kw
9. #1 OR #2 OR #3 OR #6
10. #7 OR #8
11. #9 AND #10

**Dentistry search**

Lines #1–10 as above, with additional dental filter:

12. [mh Dentistry] OR [mh "Dental Records"] OR [mh Orthodontics] OR [mh Periodontics] OR [mh Endodontics] OR [mh "Surgery, Oral"]
13. (dentistry OR dental OR dentist* OR orthodontic* OR periodontic* OR endodontic* OR "oral health" OR "oral surgery" OR maxillofacial OR "dental hygienist" OR "dental hygienists"):ti,ab,kw
14. #12 OR #13
15. #11 AND #14
